# Characterization of Tumor Antigens from Multi-omics Data: Computational Approaches and Resources

**DOI:** 10.1093/gpbjnl/qzaf001

**Published:** 2025-01-20

**Authors:** Yunzhe Wang, James Wengler, Yuzhu Fang, Joseph Zhou, Hang Ruan, Zhao Zhang, Leng Han

**Affiliations:** MOE Key Laboratory of Metabolism and Molecular Medicine, Department of Biochemistry and Molecular Biology, School of Basic Medical Sciences, Fudan University, Shanghai 200032, China; Center for Epigenetics and Disease Prevention, Institute of Biosciences and Technology, Texas A&M University, Houston, TX 77030, USA; MOE Key Laboratory of Metabolism and Molecular Medicine, Department of Biochemistry and Molecular Biology, School of Basic Medical Sciences, Fudan University, Shanghai 200032, China; Center for Epigenetics and Disease Prevention, Institute of Biosciences and Technology, Texas A&M University, Houston, TX 77030, USA; Jiangsu Key Laboratory of Infection and Immunity, Institutes of Biology and Medical Sciences, Soochow University, Suzhou 215123, China; MOE Key Laboratory of Metabolism and Molecular Medicine, Department of Biochemistry and Molecular Biology, School of Basic Medical Sciences, Fudan University, Shanghai 200032, China; Center for Epigenetics and Disease Prevention, Institute of Biosciences and Technology, Texas A&M University, Houston, TX 77030, USA; Brown Center for Immunotherapy, School of Medicine, Indiana University, Indianapolis, IN 46202, USA; Department of Biostatistics and Health Data Science, School of Medicine, Indiana University, Indianapolis, IN 46202, USA

**Keywords:** Antigen, Tumor, Multi-omics, Computational approach, Resource

## Abstract

Tumor-specific antigens, also known as neoantigens, have potential utility in anti-cancer immunotherapy, including immune checkpoint blockade (ICB), neoantigen-specific T cell receptor-engineered T (TCR-T), chimeric antigen receptor T (CAR-T), and therapeutic cancer vaccines (TCVs). After recognizing presented neoantigens, the immune system becomes activated and triggers the death of tumor cells. Neoantigens may be derived from multiple origins, including somatic mutations (single nucleotide variants, insertions/deletions, and gene fusions), circular RNAs, alternative splicing, RNA editing, and polymorphic microbiomes. An increasing amount of bioinformatics tools and algorithms are being developed to predict tumor neoantigens derived from different sources, which may require inputs from different multi-omics data. In addition, calculating the peptide–major histocompatibility complex (MHC) affinity can aid in selecting putative neoantigens, as high binding affinities facilitate antigen presentation. Based on these approaches and previous experiments, many resources have been developed to reveal the landscape of tumor neoantigens across multiple cancer types. Herein, we summarize these tools, algorithms, and resources to provide an overview of computational analysis for neoantigen discovery and prioritization, as well as the future development of potential clinical utilities in this field.

## Graphical abstract

**Figure:**
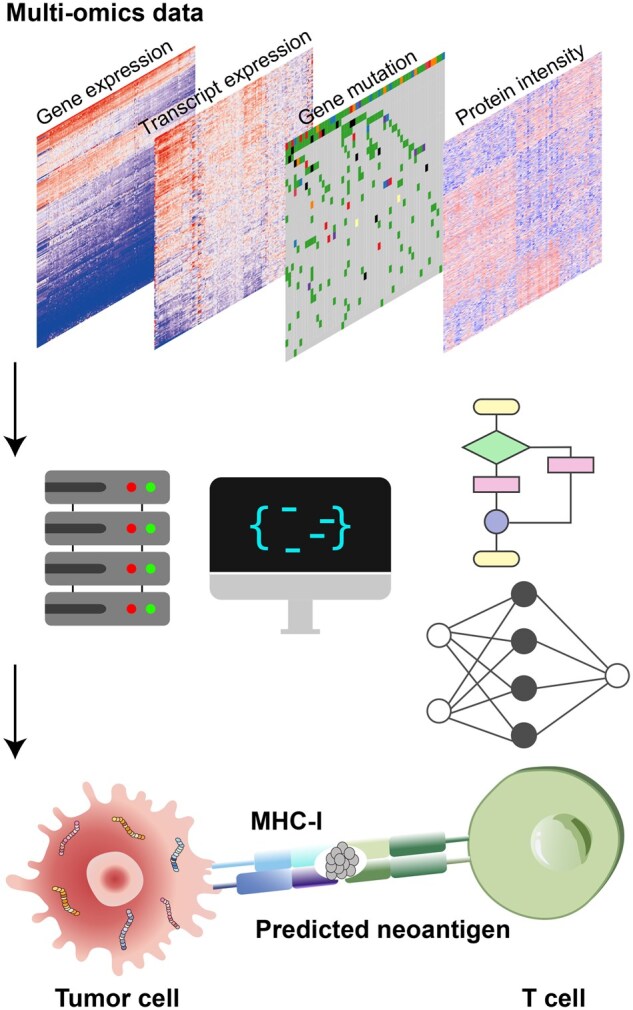

## Introduction

The adaptive immune system, with custom-tailored receptors, evolved to provide specific and flexible responses, immunologic memory, and rapid reactions upon re-exposure [1]. Cell-mediated immunity and humoral immunity work together to form the adaptive immune system, in which antigen presentation (AP) and recognition are fundamental processes. Antigens — molecules with immunogenic properties — originate from two main sources: foreign pathogens and autoantigens originating within the body [2]. Lurquin et al. demonstrated that cancerous cells present altered antigens that are not regularly found on non-cancerous cells, defined as “neoantigens” [3,4]. Neoantigens were widely detected across multiple cancer types and derived from aberrant alternations in somatic mutations, circular RNAs (circRNAs), alternative splicing (AS), RNA editing, and polymorphic microbiomes. The uniqueness of neoantigens would make cancer cells more targetable in anti-cancer therapy.

The presenting efficiency of tumor neoantigens is greatly determined by the high heterogeneity of major histocompatibility complexes (MHCs), also known as human leukocyte antigens (HLAs) in humans [5,6]. Each MHC is a heterodimer of an α and a β polypeptide chain: α chains give MHCs great potential to bind to different peptides, and β chains serve as the stabilizers of MHC structures [7–9]. Generally, MHC molecules can be divided into two classes, MHC-I and MHC-II. MHC-I molecules, encompassing HLA-A, HLA-B, and HLA-C in humans, are expressed in all nucleated cells and platelets, and present peptides derived from cytoplasmic proteins [10,11]. Within HLA-I groups, polymorphic alleles of HLAs introduce complexities into the AP process and subsequent immune responses by performing allele-specific preferences [11–13]. For example, allele HLA-A2 shares peptide specificity with aliphatic hydrophobic residues at the C-terminus [12]. Class II molecules are categorized by different chain alleles, including HLA-DP, HLA-DQ, HLA-DR, HLA-DX, HLA-DM, HLA-DOA, and HLA-DOB in humans. The contributors of polymorphism are different across these alleles. For example, the DR α chain is largely conserved, but the DQ locus contains an extensively polymorphic α chain gene [14]. HLA-II molecules present peptides derived from extracellular sources that have undergone absorption into endosomes or lysosomes [10,15].

After the AP process, MHC molecules can be recognized by T cell receptors (TCRs) on the surface of T cells, resulting in cell fate determination: the cell either remains stable or undergoes apoptosis [16]. CD8^+^ T cells recognize autologous peptides from 8 to 11 amino acids presented by MHC-I molecules. If the peptides are identified as “self” signals, T cells stay tolerant [10,17]. However, mutant sequences or microbe-derived peptides, once presented to the cell surface, can be recognized by CD8^+^ T cells as abnormal (or “non-self”) signals. These CD8^+^ T cells can then become activated and exert cytolytic functions [10]. Differently, MHC-II molecules on antigen presentation cells (APCs) present antigens to naïve CD4^+^ T cells. The recognition activates CD4^+^ T cells and transforms them into effector T cells. Subsequently, MHC-II molecules mediate interactions between B cells and macrophages with these newly activated antigen-specific CD4^+^ effector T cells [18]. Accumulated studies have focused on the impacts of utilizing neoantigen-induced immune responses against cancer, which may aid in cancer treatment.

## Clinical utility of neoantigens in cancer treatment

**Figure 1:**
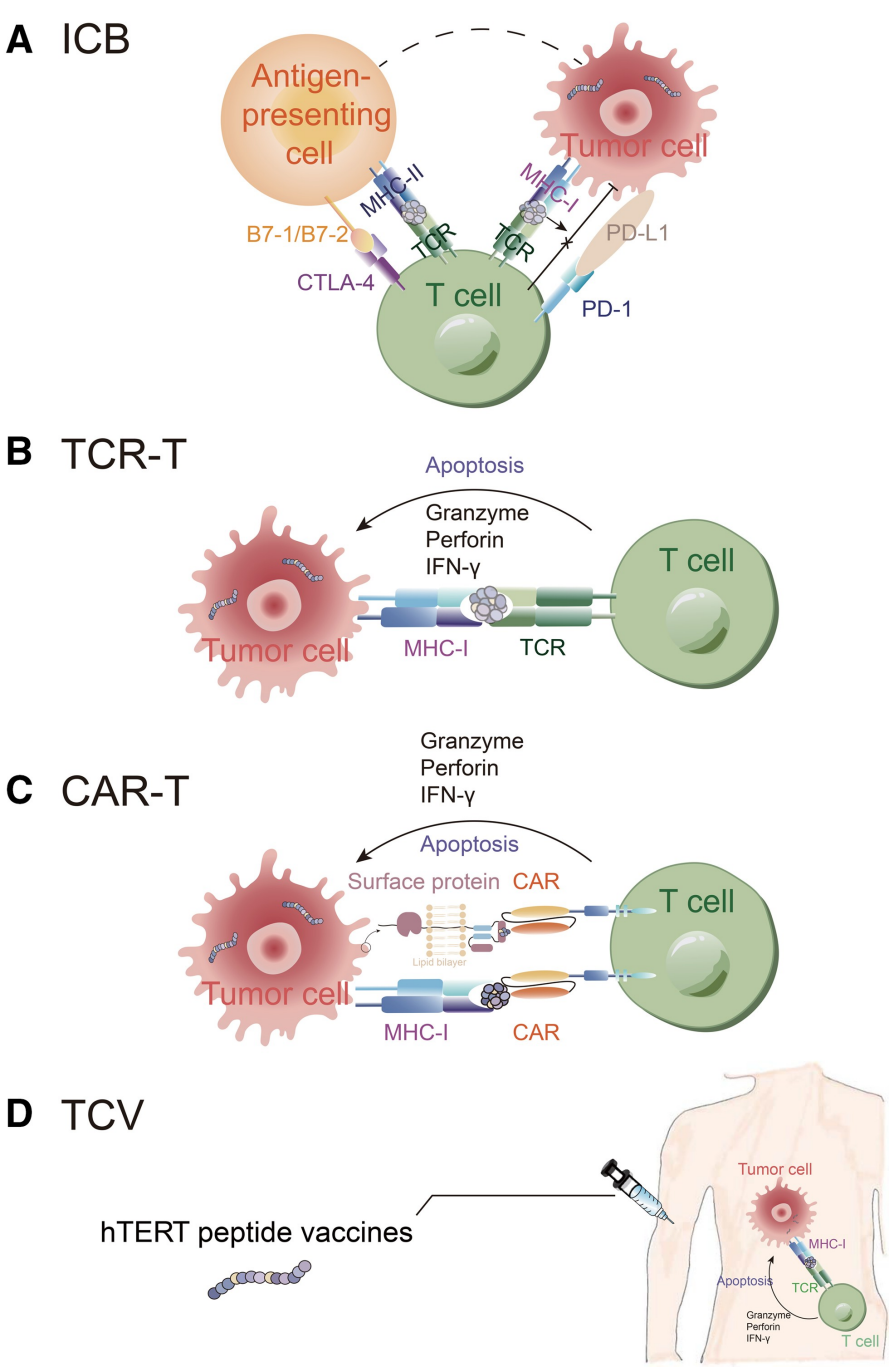
Tumor neoantigens in anti-cancer therapy **A**. Neoantigens in ICI treatment. **B**. Neoantigens in TCR-T. **C**. Neoantigens in CAR-T. **D**. Neoantigens in TCVs. ICB, immune checkpoint blockade; ICI, immune checkpoint inhibitor; TCR-T, T cell receptor-engineered T; CAR-T, chimeric antigen receptor T; TCV, therapeutic cancer vaccine; TCR, T cell receptor; CAR, chimeric antigen receptor; MHC, major histocompatibility complex; PD, programmed cell death protein; CTLA, cytotoxic T lymphocyte-associated antigen; IFN, interferon; hTERT, human telomerase reverse transcriptase.

Recent clinical endeavors in utilizing neoantigens include immune checkpoint blockade (ICB) responses, neoantigen-specific T cell receptor-engineered T (TCR-T) and chimeric antigen receptor T (CAR-T) therapies, and therapeutic cancer vaccines (TCVs) (**Figure 1**). In personalized cancer immunotherapies, neoantigens can also serve as predictors or adjuvants for ICB responses [19]. Immune checkpoint inhibitors (ICIs), such as anti-programmed cell death protein 1 (anti-PD-1) and anti-cytotoxic T lymphocyte-associated antigen 4 (anti-CTLA-4), are drugs designed to block these checkpoints, allowing the immune system to recognize and attack cancer cells more effectively [20,21].

Due to immunogenic properties, neoantigens may improve responses toward ICBs. For example, frameshift mutations generated by insertions/deletions (InDels) could produce neoantigens in non-small cell lung cancer (NSCLC), resulting in increased infiltration of activated T cells and predicting prolonged progression-free survival [22] (Figure 1A). Recent studies also found that patients with a sustained response to anti-PD-1 showed diverse neoantigen-specific T cell responses in melanoma and lung cancers [23–25]. Furthermore, neoantigens can serve as ideal targets for TCR-based immunotherapy [26]. For example, a patient with progressive metastatic pancreatic cancer was treated with autologous T cells expressing HLA-C*08:02-restricted TCRs targeting the neoantigen derived from mutant KRAS G12D, resulting in the recovery of other immune cells and regression of metastatic lung lesions [27] (Figure 1B).

CAR-T therapies genetically engineer autologous T cells by fusing an antigen-specific single-chain variable fragment (scFv) with TCR to form the chimeric antigen receptor (CAR) [28–30]. Traditional CAR-T therapies recognize antigens through HLA-independent and antibody-like-mediated processes [31]. For example, anti-GD2 CAR T cells can target glioma cells expressing K27M mutation in the gene of histone H3 (H3K27M-mutated glioma cells), thus performing antigen-specific cytotoxicity [32]. Recent studies also designed scFvs to target neoantigen–HLA complexes to enhance immunological responses [33,34]. For example, CAR-T cells target a nucleophosmin-derived neoantigen, NPM1c, which could be presented by HLA-A2 in patients with acute myeloid leukemia, thus mediating anti-tumor immunity [34] (Figure 1C).

In addition, neoantigens can potentially be engineered as TCVs in therapy [35–37]. A peptide vaccine derived from the reverse transcriptase subunit of telomerase (hTERT) has been shown to induce immune responses in metastatic hormone-naïve prostate cancer [38] (Figure 1D). Some neoantigen-based TCV platforms have also been put into clinical trials (NCT02348320 and NCT03122106) to enhance immune responses against cancer [39,40].

Mining potential neoantigens from multi-omics data could provide effective information for clinical utility. The origins and immunogenicity of potential neoantigens, the types of HLA alleles, and the binding affinity between them all affect the *in silico* prediction of neoantigens. Therefore, these characteristics should be carefully considered when developing tools and algorithms. Using these tools and algorithms, neoantigens could be identified across various cancer types.

## Tools and algorithms for predicting neoantigens from different origins

The current landscape of bioinformatics tools and algorithms designed for neoantigen prediction exhibits diverse techniques. They generally share a common workflow to streamline the identification process [41]. This typical workflow involves several key steps: (1) identification of neoantigen candidates: extraction of putative neoantigen candidates from the input genomic, transcriptomic, or proteomic data, considering different origins of neoantigens; (2) detection of HLA alleles: determination of the patient’s HLA alleles, providing essential information for subsequent analyses; (3) prediction of peptide–HLA binding affinity and stability: assessment of the binding affinity and stability of the identified neoantigen candidates with the patients’ HLA molecules, ensuring compatibility for effective presentation to immune cells; (4) prioritization of neoantigen candidates: selection and prioritization of the most promising neoantigen candidates based on various criteria, such as predicted immunogenicity, likelihood of eliciting immune responses, and expression validations [39]. These computational approaches hold the promise of unlocking the potential of neoantigens in personalized cancer treatment.

Neoantigens may arise from multiple origins, which must be considered in the development of tools and algorithms. Somatic mutations, referring to alterations at the cellular level in somatic tissues occurring after fertilization, have long been regarded as the most frequent origin of neoantigens. Among mutations, single nucleotide variants (SNVs) and InDels could generate aberrant open reading frames (ORFs), which may be transcribed and translated into neoantigens. Another type of somatic mutation, fusion genes, can also generate neoantigens due to the erroneous fusions of different sequences. Other abnormal transcriptional and/or post-transcriptional events, including circRNAs, AS, and RNA editing, can also produce neoantigens, especially when the RNAs contain and alter ORFs within their sequences. Additionally, the polymorphic microbiome, recently listed as one of the hallmarks of cancer, is likely to be lysed into peptides serving as neoantigens. These highly specific neoantigens from different origins necessitate individualized identification for each patient [42]. Experimental breakthroughs incorporating identified neoantigens have shown anti-tumoral effects across cancer types. In parallel, the field of computational-level identification of neoantigens has also experienced rapid advancements in recent years. The computational approach is crucial in providing a systematic and efficient means of predicting neoantigens that can selectively stimulate T cell responses to target and eliminate cancer cells.

### Predicting neoantigens from somatic mutations

Somatic mutations are spontaneously occurring mutations that accumulate in somatic cells, including SNVs, InDels, copy number variations (CNVs), and gene fusions [43,44]. In this section, we review the tools focusing on SNVs, InDels, and fusion genes, as these types of mutations are more likely to produce aberrant peptide sequences with AP potentials. As tumors with a mutation load above ten somatic mutations per megabase (Mb) tend to share common formations of neoantigens potentially recognized by T cells, somatic mutations are thought to be the primary origin of neoantigens [35]. A diverse array of tools has been developed to automate these processes.

#### Predicting neoantigens from SNVs and InDels

**Figure 2:**
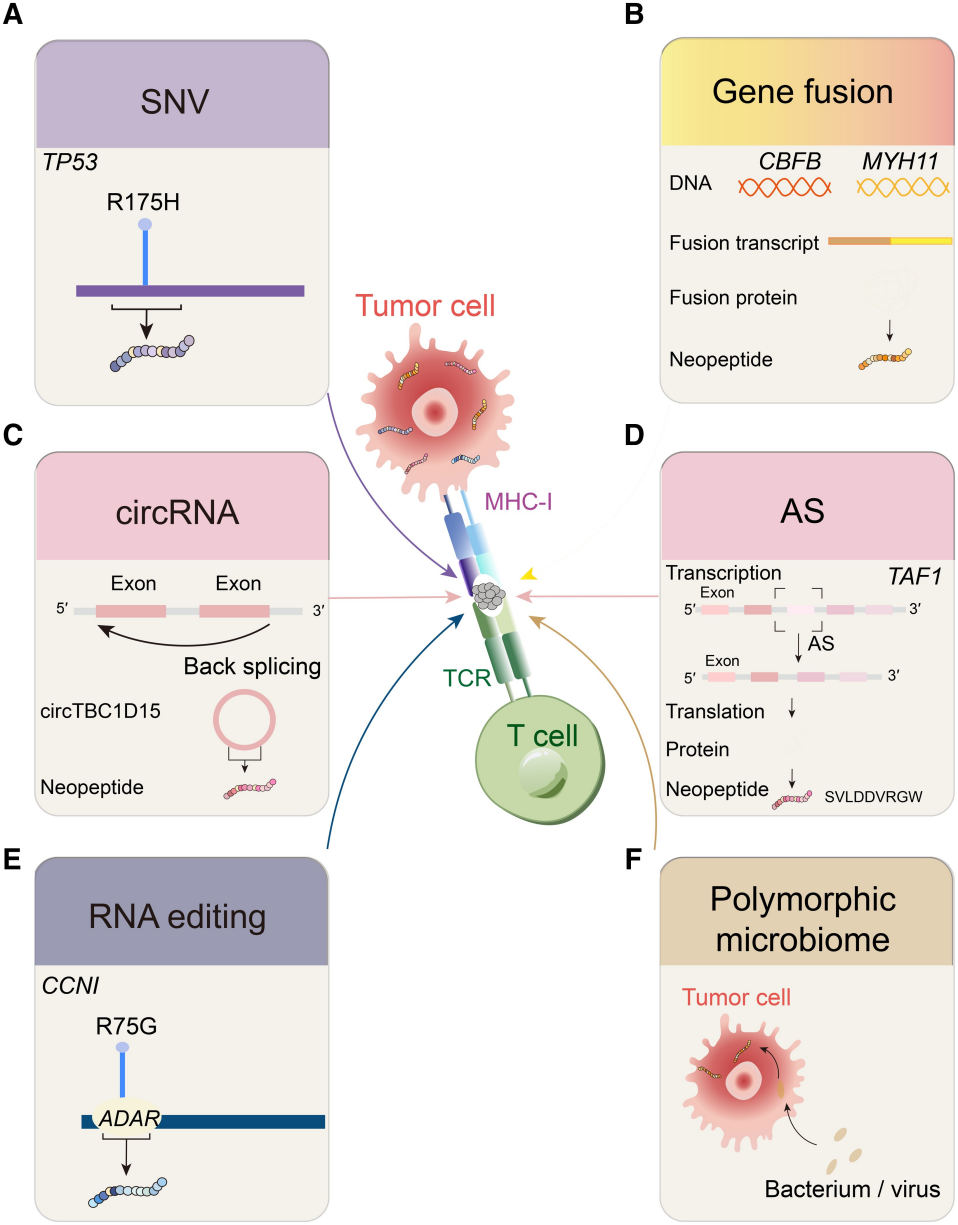
Diverse origins of tumor neoantigens **A**. Neoantigens derived from SNVs, taking the R175H mutation in *TP53* as an example. **B**. Neoantigens derived from gene fusions, taking *CBFB*–*MYH11* fusion as an example. **C**. Neoantigens derived from circRNAs, taking circTBC1D15 as an example. **D**. Neoantigens derived from AS events, taking SVLDDVRGW from the *TAF1* gene in leukemia cell line HL-60 as an example. **E**. Neoantigens derived from RNA editing events, taking the edited peptide from *CCNI* as an example. **F**. Neoantigens derived from polymorphic microbiomes, including bacteria and viruses. circRNA, circular RNA; SNV, single nucleotide variant; AS, alternative splicing.

SNVs and InDels may result in nonsynonymous mutations and/or novel ORFs that alter the amino acid sequences and generate abnormal peptides [45–48]. For example, explicitly targeting the neoantigen derived from the most common *TP53* mutation, R175H, in complex with HLA-A has been proved to effectively activate T cells, leading to the lysis of cancer cells by presenting the neoantigen *in vitro* and in mice [49] (**Figure 2**A). More importantly, neoantigens derived from mutant *AIM2*, *HT001*, and *TAF1B* have already undergone clinical trials, providing promising prospects for utilizing somatic mutation-derived neoantigens [50].

The workflow for targeting SNVs and InDels usually takes two types of inputs: (1) genome sequencing data of paired tumor and normal samples and (2) pre-analyzed mutation files (also derived from genome sequencing) [47,51]. In **Table 1**, we summarize the tools capable of predicting neoantigens originating from SNVs and InDels. Some tools specifically predict neoantigens from SNVs and InDels, such as personalized variant antigens by cancer sequencing (pVAC-Seq), tumor-specific neoantigen detector (TSNAD), prioritizing tumor neoantigens (pTuneos), Neopepsee, PGNneo, neoantigen prediction pipeline (NeoPredPipe), mutant peptide extractor and informer (MuPeXI), CloudNeo, TIminer, and proteogenomics prediction of neoantigen (ProGeo-neo) [52–61]. Most tools only take sequencing data as input; however, PGNneo requires mass spectrometry (MS) data as input to further confirm the existence of candidate neoantigens at the proteomic level [56,62]. On the other hand, some tools can predict neoantigens derived from different origins (*e.g.*, fusion genes, AS, and/or microbiome, see below), such as landscape of effective neoantigens software (LENS), ScanNeo2, ProGeo-neo v2.0, nextflow neoantigen prediction pipeline (nextNEOpi), TruNeo, TSNAD v2.0, and Seq2Neo [63–69]. The multiple origins provided by these tools also require different sorts of omics data.

**Table 1:** Bioinformatics tools for predicting neoantigens

Tool	Input omics	Origin type	Origin prediction	HLA typing	Binding prediction	Neoantigen prioritization	System	Language	Link	Ref.
LENS	Genome, transcriptome	SNVs/InDels, gene fusions, AS, viruses	GATK Mutect2 [165], Strelka2 [166], ABRA2 [167], NeoSplice [103], STAR-Fusion [168], VirDetect [131]	NA	NetMHCpan-4.0 [94], NetMHCstabpan [143], MHCflurry [140], DeepHLApan [141], atigen.garnish [169]	Harmonization metric [63]	Linux	Python	https://gitlab.com/landscape-of-effective-neoantigens-software	[63]
ScanNeo2	Genome, transcriptome	SNVs/InDels, gene fusions, AS	GATK Mutect2 [165], transIndel [170], SplAdder [171], ScanExitron [162]	OptiType [133], HLA-HD [135]	NetMHC [138], NetMHCpan-4.0 [116], NetMHCII [139], NetMHCIIpan-3.0 [172]	IEDB immunogenicity [144]	Linux	Python	https://github.com/ylab-hi/ScanNeo2/	[64]
ProGeo-neo v2.0	Genome, transcriptome, proteome	SNVs/InDels, gene fusions	GATK Mutect2 [165], STAR-Fusion [168]	OptiType [133], HLAminer [136]	NetMHCpan-4.1 [94], NetMHCIIpan-4.0 [116]	Expression validation [65], BLASTP [147]	Linux	Python	https://github.com/kbvstmd/ProGeo-neo2.0	[65]
nextNEOpi	Genome, transcriptome	SNVs/InDels, gene fusions	pVACseq [52], NeoFuse [77]	OptiType [133], HLA-HD [135]	NetMHCpan-4.0 [116], MHCflurry [140]	MiXCR [145]	Linux	Nextflow, R, Python	https://github.com/icbi-lab/nextNEOpi	[66]
TruNeo	Genome, transcriptome	SNVs/InDels, gene fusions	VarScan 2 [173], GATK somatic InDel detector [165], STAR-Fusion [168]	Polysolver [134], BWA-HLA	NetMHCpan-3.0 [137], MHCflurry [140]	Score combining VAF [67], BA [67], TPM [67]	Linux	Perl, Python	https://github.com/yucebio/TruNeo	[67]
TSNAD v2.0	Genome, transcriptome	SNVs/InDels, gene fusions	GATK Mutect2 [165], Arriba [174]	OptiType [133]	DeepHLApan [141]	Binding score [68], immunogenic score [68], expression validation [68]	Linux	Perl, Python	https://github.com/jiujiezz/tsnad	[68]
Seq2Neo	Genome, transcriptome	SNVs/InDels, gene fusions	GATK Mutect2 [165], STAR-Fusion [168]	HLA-HD [135]	NetMHCpan-4.0 [116]	Seq2Neo-CNN [69]	Linux	Python	https://github.com/XSLiuLab/Seq2Neo	[69]
pVAC-Seq	Genome, transcriptome	SNVs/InDels	VarScan 2 [173]	HLAminer [136], ATHLATES [175]	NetMHC 3.4 [138]	MT binding score [52], bam-readcount [52], expression validation [52]	Linux, MacOS, Windows	Python	https://github.com/griffithlab/pVAC-Seq	[52]
TSNAD	Genome, transcriptome	SNVs/InDels	GATK Mutect2 [165]	SOAP-HLA [176]	NetMHCpan-2.8 [177]	NA	Linux	Python, Perl	https://github.com/jiujiezz/tsnad	[178]
pTuneos	Genome, transcriptome	SNVs/InDels	GATK Mutect2 [165], VarScan 2 [173], Strelka2 [166]	OptiType [133]	NetMHCpan-4.0 [116]	MHC multimer analysis [179]	Linux	Python	https://github.com/bm2-lab/pTuneos	[54]
Neopepsee	Genome, transcriptome	SNVs/InDels	NA	NA	NetCTLpan [180]	Immunogenicity classifier [55]	Windows	Java	https://sourceforge.net/projects/neopepsee/	[55]
PGNneo	Transcriptome, proteome	SNVs/InDels	GATK Mutect2 [165]	OptiType [133]	NetMHCpan-4.1 [94]	BLASTP [150]	Linux	Python, R	https://github.com/tanxiaoxiu/PGNneo	[56]
NeoPredPipe	Genome, transcriptome	SNVs/InDels	NA	NA	NetMHCpan-4.0 [116]	Peptide Match [148], immune fitness model [146]	Linux	Python, R	https://github.com/MathOnco/NeoPredPipe	[57]
MuPeXI	Genome, transcriptome	SNVs/InDels	GATK Mutect2 [165]	NA	NetMHCpan-4.0 [116]	Priority score [59]	Linux, MacOS	Python	https://github.com/ambj/MuPeXI	[58]
CloudNeo	Genome, transcriptome	SNVs/InDels	NA	Polysolver [134], HLAminer [136]	NetMHCpan-3.0 [137]	NA	Linux, MacOS, Windows	Common workflow language	https://github.com/TheJacksonLaboratory/CloudNeo	[59]
TIminer	Genome, transcriptome	SNVs	NA	OptiType [133]	NetMHCpan-3.0 [137]	Expression validation [60]	Linux, MacOS	Python	https://icbi.i-med.ac.at/software/timiner/timiner.shtml	[60]
ProGeo-neo	Genome, transcriptome, proteome	SNVs	BCFtools [181]	OptiType [133]	NetMHCpan-4.0 [116]	Expression validation [61], BLASTP [147], OmicsBean-Cancer workflow [61], SAAV mapping [61]	Linux	Python	https://github.com/kbvstmd/ProGeo-neo	[61]
INTEGRATE-Neo	Genome, transcriptome	Gene fusions	INTEGRATE [182]	HLAminer [136]	NetMHC 4.0 [138]	NA	Linux	C++, Python	https://github.com/ChrisMaherLab/INTEGRATE-Neo	[76]
NeoFuse	Transcriptome	Gene fusions	Arriba [174]	OptiType [133]	MHCflurry [140]	IC50 [77], confidence score [77]	Linux, MacOS	Shell, Python	https://github.com/icbi-lab/NeoFuse	[77]
neoFusion	Proteome	Gene fusions	neoFusion search	NA	NetMHCpan-4.0 [116]	Target-decoy based FDR [78]	Linux	Python	https://github.com/bm2-lab/neoFusion	[78]
pVACfuse	Transcriptome	Gene fusions	AGFusion [183], Arriba [174], STAR-Fusion [168]	IEDB RESTful [150]	NetMHCpan-3.0 [137], NetMHC [138], NetMHCcons [184], PickPocket [185], SMM-align [186], SMMPMBEC [187], MHCflurry [140], NetMHCIIpan-3.0 [172], NN-align [188]	NetChop [189], BLASTP [147]	Linux, MacOS, Windows	Python, Perl	https://pvactools.readthedocs.io/en/latest/pvacfuse.html	[79]
CIRC_neo	Transcriptome	circRNA	find_circ, CIRCexplorer2 [190], IRESfinder [191], CPAT [92], CPC2 [192]	OptiType [133], HLA-HD [135]	NetMHCpan-4.0 [116], MixMHC2pred [193]	NetChop [189]	NA	Python, R	https://github.com/summerjiaqi/CIRC_neo	[82]
CICADA	Transcriptome, proteome	circRNA	CICADA	NA	NetMHCpan-4.1 [94]	NA	Linux	Python, R	https://github.com/SunLab-biotool/CICADA	[88]
SNAF	Transcriptome	AS	AltAnalyze [194]	OptiType [133]	NetMHCpan-4.1 [94], MHCflurry [140]	DeepImmuno [195], BayesTS [196]	Linux	Python, Shell	https://github.com/frankligy/SNAF	[197]
Retained-intron-neoantigen-pipeline	Genome, transcriptome	AS	Retained-intron-neoantigen-pipeline	Polysover [134]	NetMHCpan-3.0 [137]	NA	Linux	Python, Shell	https://github.com/vanallenlab/retained-intron-neoantigen-pipeline	[101]
ASNEO	Genome, transcriptome, proteome	AS	STAR [198]	OptiType [133]	NetMHCpan-4.0 [116]	Immune score [102]	Linux	Python	https://github.com/bm2-lab/ASNEO	[102]
NeoSplice	Transcriptome	AS	BWT-based algorithm, depth-first search, graph traversal	OptiType [133]	NetMHCpan-4.0 [116]	NA	Linux	Emacs Lisp	https://github.com/Benjamin-Vincent-Lab/NeoSplice	[103]
IRIS	Transcriptome, proteome	AS	STAR [198], rMATS [199]	seq2HLA [200]	IEDB predictor [150]	PSI-based screening [104], SJC-based screening [104]	Linux	Python	https://github.com/Xinglab/IRIS	[104]
SpliceMutr	Transcriptome	AS	STAR [198], LeafCutter [201]	arcasHLA [202]	MHCnuggets [203]	Splicing antigenicity metric [105]	Linux	R, Python	https://github.com/FertigLab/splicemute	[105]

*Note*: SNV, single nucleotide variant; InDel, insertion/deletion; circRNA, circular RNA; AS, alternative splicing; NA, not available.

Though the tools exhibit shared workflows, they may have tool-specific processes. Taking pVAC-Seq as an example, it requires properly formatted lists of annotated variants explaining the amino acid changes and transcript sequences to predict binding affinity. Specifically, differences between tumor and normal peptides are compared to enhance the prediction performance. Isoform-level expression information from RNA sequencing (RNA-seq) is also incorporated to accurately exclude variants that are not actively expressed in tumor cells. Altogether, multi-omics data and information from different aspects are analyzed in the pipeline of pVAC-Seq [52]. Importantly, neoantigens identified by pVACtools were designed as TCVs using a newly developed DNA vaccine platform. On average, each vaccine contained 11 neoantigens per patient and was then put into clinical trial (NCT02348320) [40]. Furthermore, another clinical trial (NCT03122106) based on pVACtools-predicted neoantigens was also conducted in pancreatic cancer patients (*n* = 15) [39]. Some tools incorporated multiple machine learning algorithms to improve the prediction of neoantigens from SNVs/InDels. For example, Neopepsee takes a list of mutations and the raw RNA-seq data as inputs and then automatically predicts neoantigens based on four machine learning models, including Gaussian naïve Bayes, locally weighted naïve Bayes, random forest, and support vector machine. After the calculation, it outputs rich annotations for candidate peptides, such as peptide half-life, peptide sequences, neoantigen probabilities in three levels, and expression levels of neoantigens with immune regulatory genes [55].

Together, these integrated pipelines and newly developed algorithms provide *in silico* prediction of neoantigens originating from SNVs and InDels according to genome data and may validate results on other omics data. Differently, the computational-level identification of fusion genes is mainly based on RNA-seq data, which makes neoantigen prediction from SNVs/InDels and fusion genes differ in inputs and processing. Therefore, we separate the prediction of fusion-derived neoantigens from SNVs/InDels and will introduce the related tools in the next section.

#### Predicting neoantigens from fusion genes

Gene fusions occur when two genes become physical neighbors in the DNA sequence due to mutations and are expressed as a singular RNA or when two adjacent genes are erroneously translated together as a single gene due to transcription read-through [70,71]. Peptides spanning the breakpoint regions of different genes compose fusion proteins that differ from self-antigens and can serve as neoantigens pending T cell recognition [72,73]. Although the formation of fusion genes is a relatively rare event compared to other somatic mutations, the neoantigens derived from fusion genes exhibit higher immunogenicity than those derived from pure mutations. This heightened immunogenicity makes fusion genes a compelling area of interest for immunotherapy development [70]. For instance, the *CBFB*–*MYH11* fusion neoantigen was found to be immunogenic and capable of enabling T cells to kill acute myeloid leukemia cells [74] (Figure 2B). In fibrolamellar carcinoma, *DNAJB1*–*PRKACA* fusion neoantigens, exhibiting similar immunogenic characteristics, have also been utilized with paired fusion–TCRs to better enhance the adoptive T cell therapies [75].

In tools and algorithms predicting fusion-derived neoantigens, RNA-seq data are requisite. Some tools, such as LENS, ScanNeo2, ProGeo-neo v2.0, nextNEOpi, TruNeo, TSNAD v2.0, and Seq2Neo, are originally designed for predicting SNV/InDel-derived neoantigens, as mentioned above. However, they also have the capability to predict neoantigens originating from fusion genes [63–69] (Table 1). After the prediction is performed, some tools also establish scoring systems to aid in selecting neoantigen candidates. For example, TruNeo incorporates deep learning models, the transport efficiency of transporter associated with antigen processing (TAP), and the peptide–MHC affinity to form a comprehensive scoring system. Candidate peptides passing the scoring system would be identified as neoantigens [67]. Other tools, such as INTEGRATE-Neo, NeoFuse, neoFusion, and pVACfuse, treat the prediction of fusion gene-derived neoantigens as an independent process [76–79]. For example, INTEGRATE-Neo first predicts gene fusion peptides and then performs neoantigen discovery within these candidate peptides based on HLA-binding affinity [76]. The ability to discern and predict neoantigens arising from fusion genes underscores the importance of these computational tools in helping researchers understand fusion events and their potential influence on cancer immunology.

### Predicting neoantigens from circRNAs

The circRNAs, single-stranded continuous loops of RNAs spliced from linear RNAs, are usually derived from back-splicing events [80]. The peptide-encoding potentials of circRNAs, if they contain position-altered ORFs within the sequences, suggest their ability to produce neoantigens [81–85]. For example, the circTBC1D15-derived neoantigen has been proved to be presented by HLA-A, HLA-B, and HLA-C, drastically reducing the survival rate of the tumor organoid [86] (Figure 2C). Advancements in RNA-seq techniques, particularly those utilizing enriched non-polyadenylated transcriptomes, make the detection of circRNAs much easier [87].

While various methods have been developed to discover circRNAs, bioinformatics tools capable of predicting immunogenic peptides derived from circRNAs remain relatively scarce. One comprehensive pipeline for predicting such neoantigens is circRNA-derived neoepitope prediction pipeline (CIRC_neo). It integrates circRNA finders, coding potential identifiers, HLA-typing methods, and binding affinity predictors to form the whole pipeline, which is designed to sift through total RNA-seq or circRNA sequencing data to identify putative neoepitopes from circRNAs [82]. Another tool, named circRNA coding capability and product detection algorithm (CICADA), employs a machine learning model to discern circRNAs with high coding capability and high HLA-binding affinity. This approach enables the prediction of neoantigen potentials arising from circRNAs [88] (Table 1). As the understanding of circRNAs and their potential immunological significance continues to deepen, these computational tools play an increasingly important role in facilitating circRNA detection and exploring their role in the intricate network of neoantigen formation. Accumulated studies demonstrated that neoantigens could also be translated from some long non-coding RNAs (lncRNAs) [89,90]. Studies have integrated current tools like ORFfinder, CPAT, OrthoMCL, and NetMHCpan with algorithms like multistate thermodynamic models to form the pipelines for detecting neoantigens from lncRNAs [91–96] (see below).

### Predicting neoantigens from AS

AS refers to a regulated process in which a strand of pre-mRNA can be variably spliced into different mature messenger RNAs (mRNAs) through adjoining different exons, which gives rise to the phenomenon wherein a singular gene encodes multiple proteins [97]. Due to the highly regulated properties of AS in different tissues, some isoforms generated from the events can serve as tissue-specific or state-specific markers. AS events mainly include five modes: exon skipping, alternative 5′ splice-site selection, alternative 3′ splice-site selection, intron retention, and mutually exclusive exons [97]. Specifically, among these modes, intron retention may arise either through a dysregulated process impacting specific junctions or incomplete processing across the entire gene [98]. The alterations and dysregulation of AS events may contribute to increased complexities of transcriptomes as well as their translated products [99,100]. For example, Smart et al. identified peptides translated from retained introns as a source of cancer-specific neoantigens, such as peptide sequence SVLDDVRGW from the *TAF1* gene in leukemia cell line HL-60 [101]. A comprehensive study focused on neoantigens derived from AS neo-junctions has also highlighted the expanded target field for immunotherapy based on pan-cancer analysis [11]. Identifying such events and utilizing abnormal proteins as neoantigens might aid in finding new immunogenic targets.

Tools predicting neoantigens from AS include LENS, retained-intron-neoantigen-pipeline, alternative splicing neoantigens (ASNEO), NeoSplice, isoform peptides from RNA splicing for immunotherapy target screening (IRIS), and SpliceMutr [63,101–105] (Table 1). IRIS provides AS-derived neoantigen predictions together with CAR-T annotations, making the results more applicable to a clinical setting [104]. Among the tools, retained-intron-neoantigen-pipeline was the first to specifically focus on intron retentions. It generates peptide sequences after identifying retained intron loci, taking both the ORF orientation and normal retention events into account [101]. A newly developed pipeline, pVACsplice, will soon be incorporated into pVACtools to further expand the comprehensive neoantigen-predicting toolkit [79]. In these tools, researchers could use tumor–normal paired samples to distinguish those AS events specifically happening in tumor tissues [106]. For example, NeoSplice requires matched tumor and normal RNA-seq data as inputs to identify isoforms specifically appearing in tumor [103]. Tools like ASNEO and splicing neo antigen finder (SNAF) use the expression of normal tissues based on Genotype-Tissue Expression (GTEx) portal data as references in case there is a lack of paired normal data [102,107].

### Predicting neoantigens from RNA editing

RNA editing, whose dysregulation is frequent across different cancer types, involves chemically modifying RNA nucleotides [108–110] (Figure 2D). One common type of RNA editing converts A to I or C to U / U to C, which could change the amino acid sequences and produce “edited” peptides with potential immunogenic properties. The conversion of A to I is the most common editing pattern, producing accordingly altered peptides [110]. Recent studies have proved that the edited peptide from *CCNI* showed elevated abundances in several tumors and was capable of stimulating T cell responses in melanoma tumors when presented by HLA-A*02:01 [111] (Figure 2E).

The conversion can be predicted by a variety of algorithms, such as RNA/DNA difference prediction (RDDpred), RNA-DNA differences with support vector machines (RDDSVM), and plant RNA editing prediction & analysis computer tool (PREPACT; v2.0) [112–114]. Some tools, like A-to-I editing predictor (ATTIC), also provide “edited” peptide sequences after predicting RNA editing events [115]. Researchers have incorporated peptide–HLA affinity predictors, like NetMHCpan, used in aforementioned pipelines to design an RNA editing neoantigen immunogenicity score schema [116,117]. However, tools combining RNA editing prediction and subsequent neoantigen prediction are still lacking, calling for further development.

### Predicting neoantigens from polymorphic microbiomes

In the latest dimensions of cancer hallmarks, polymorphic microbiomes have been regarded as new hallmarks [118]. Due to the dysfunctional status of tumor cells, various types of microbes may enter tumor cells to produce “non-self” proteins [119–122]. An example of such proteins is viral oncoproteins, defined as viral proteins with oncogenic properties and common in viral infection-induced cancers, such as cervical cancers derived from human papillomaviruses (HPVs) [123–125]. As typical pathogens markedly differ from human peptides, viral antigens tend to have a high affinity with TCRs [126]. Similar to viral oncoproteins, intratumoral bacteria and the gut microbiome can also produce specific peptides (Figure 2F). For example, neoantigen pACP1780 and pACP2283, discovered from gut microbiome-derived peptides, have been proved to inhibit tumor growth both *in vivo* and in mice [127]. In addition, some engineered bacteria, such as *Escherichia coli* genetically engineered to carry bacteria derived vesicle-neoantigen (BDVs-Neo), have also shown their potency in producing specific neoantigens and eliciting systemic anti-tumor immunity after entering host cells [128,129]. The discovery of the intratumor microbiome, spanning a diverse range of cancer types, has unveiled its dual functions in influencing cancer progression [122,130]. Some microbiome-derived proteins may be lysed to oligopeptides and presented by HLAs on tumor cell surface. Based on their exogenous characteristics, tools and algorithms predicting microbiome-derived neoantigens could skip the processes of identifying changed ORFs and immunogenic properties. Accordingly, assigning sequences to different microbial taxonomies becomes a main upstream process in predicting such neoantigens.

Among the aforementioned algorithms, LENS incorporates VirDetect to detect viral sequences from RNA-seq data [63,131]. Additionally, it uses BCFtools to detect homozygous germline variants and generate viral peptides. After the peptide has been identified, the binding affinity and stability can be tested to finally select neoantigens from candidate peptides [63]. Another tool, pVAC-Seq, originally developed to predict neoantigens from somatic mutations, has also demonstrated its potential utility in predicting bacterial and/or viral neoantigens [52,132]. Tools like ProGeo-neo have taken a comprehensive approach by including bacterial and viral peptides in their consideration of immunogenic HLA-binding peptides during prediction processes [61] (Table 1). Despite these advancements, it is noteworthy that with our current knowledge, there is a lack of specific tools or algorithms predicting intratumor bacteria-derived neoantigens, indicating a necessity for future research and tool development.

## Incorporated tools and algorithms aiding in neoantigen prediction

HLA typing is usually the primary step for sample-specific neoantigen–HLA binding predictions (Table 1). OptiType and Polysolver are widely used for predicting HLA-I alleles; HLA-HD and HLAminer are similarly popular for predicting HLA-II alleles [133–136]. For neoantigen prediction tools that don’t include HLA identification, they either require users to provide clinical files of sample-specific HLA alleles or use the most frequent HLA alleles among the human populations [57,58].

**Figure 3:**
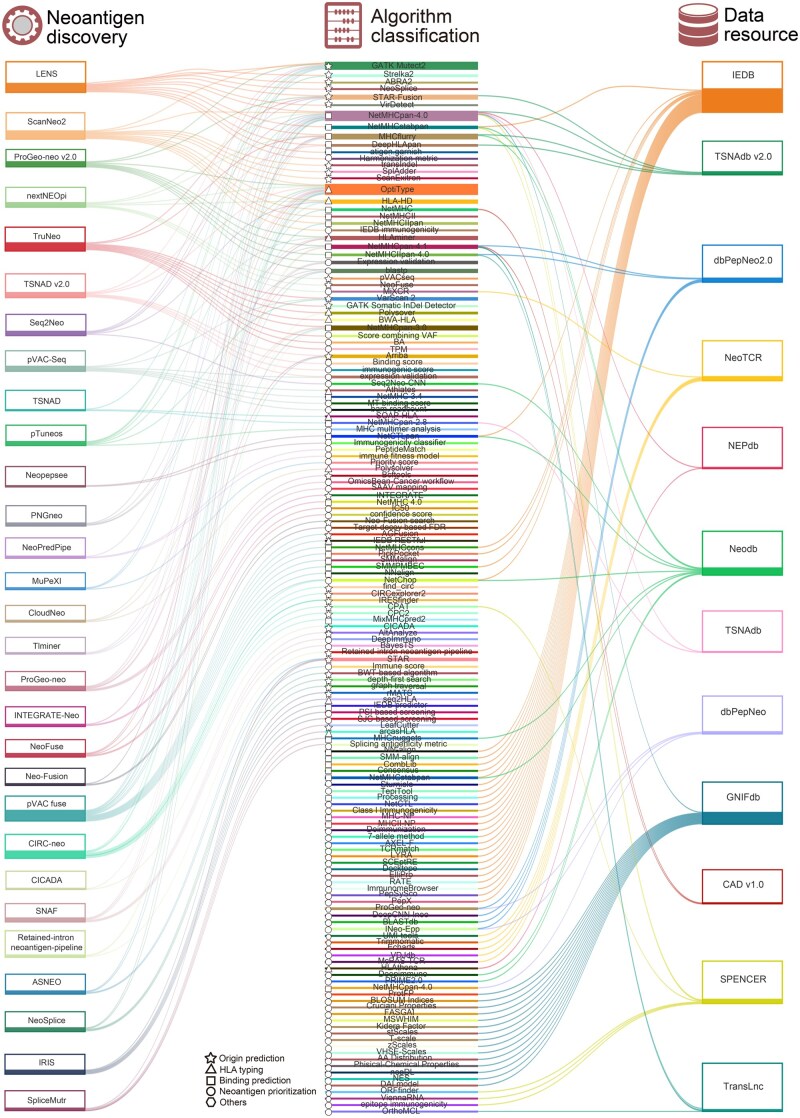
Summary of neoantigen discovery tools, affinity prediction methods, and neoantigen data portals The figure illustrates the methods (middle bars) shared by neoantigen discovery tools (left bars) and databases (right bars). Symbols denote origin prediction (star), HLA typing (triangle), binding prediction (rectangle), neoantigen prioritization (circle), and others (hexagon). HLA, human leukocyte antigen; LENS, landscape of effective neoantigens software; nextNEOpi, nextflow neoantigen prediction pipeline; pVAC-Seq, personalized variant antigens by cancer sequencing; TSNAD, tumor-specific neoantigen detector; pTuneos, prioritizing tumor neoantigens; NeoPredPipe, neoantigen prediction pipeline; MuPeXI, mutant peptide extractor and informer; ProGeo-neo, proteogenomics prediction of neoantigen; CIRC_neo, circRNA-derived neoepitope prediction pipeline; CICADA, circRNA coding capability and product detection algorithm; ASNEO, alternative splicing neoantigens; SNAF, splicing neo antigen finder; GTEx, Genotype-Tissue Expression; TSNAdb, tumor-specific neoantigen database; IEDB, immune epitope database and analysis resources; CAD, cancer antigens database; SPENCER, small peptides encoded by ncRNA from cancer patients.

Predicting the binding affinity of peptide–MHC is a critical step in the selection and determination of candidate neoantigens, which decides whether these predicted peptides could be presented as antigens by HLAs. Almost all the tools for neoantigen discovery incorporated at least one algorithm to predict the binding affinity. Indeed, in the workflow of neoantigen predictors, 19 algorithms were included and shared by them, such as NetMHCpan, NetMHC, NetMHCII, and MHCflurry [116,94,137–140] (**Figure 3**; Table 1). Machine learning methods or neural networks are essential in these algorithms, in which MS data and/or sequencing data are included as weights for training processes to enhance the prediction performance of binding affinity scores. NetMHCpan, one of the most popular algorithms, was incorporated in 22 listed tools (Figure 3; Table 1). NetMHCpan performs artificial neural networks (ANNs) on the binding affinity values and MS eluted ligand data to build its model. With any given HLA typing or sequence with candidate peptides, it can predict allele-specific affinity scores based on this model, which can be used as an important filter excluding those peptides exhibiting low affinities toward HLA alleles [116]. Another commonly used algorithm is MHCflurry, incorporated in 7 listed tools (Figure 3; Table 1). To improve prediction performance, it separates the predictors into two categories: one for MHC allele-dependent effects (binding affinity prediction) and the other for allele-independent effects (AP prediction), and then combines the two predictors in a logistic regression model. Any MHC-I alleles with known sequences could be predicted by MHCflurry based on the combined predictor [140]. DeepHLApan, incorporated in TSNAD v2.0 and tumor-specific neoantigen database (TSNAdb; v2.0), combines predicting peptide–MHC binding affinity and immunogenicity together, thus providing more options for neoantigen selection [68,141,142]. Additionally, some tools mainly target the affinity between peptide sequences and MHC-II alleles. Taking NetMHCII as an example, it could perform predictions on 25 HLA-DR, 20 HLA-DQ, 9 HLA-DP, and 7 mouse H2 class II alleles once the peptide sequences are provided [139]. Besides the binding affinity, binding stability is also important for a peptide to be translocated from MHCs to cytotoxic T lymphocytes (CTLs), thus inducing an immune response. This type of algorithm, such as NetMHCstabpan, is also incorporated into some tools [143]. NetMHCstabpan has been trained on quantitative stability data and uses ANNs to build its predictor. Similar to NetMHCpan, given any HLA typing or sequence with the candidate peptides, it can provide the stability score of putative neoantigens [116,143]. With the binding affinity and stability predicted, the possibility of the AP process can be determined *in silico*. Peptides with high binding affinities and stabilities to HLA molecules are prioritized.

There are various criteria to prioritize neoantigen candidates. Immunogenicity, potential immune responses, and expression validation are widely considered (Table 1). Different methods may define their own criteria based on these aspects. Immunogenicity can be predicted by tools like immune epitope database and analysis resources (IEDB) immunogenicity and DeepHLApan [141,144]. Immune responses can be predicted by tools (*e.g.*, MiXCR) and algorithms (*e.g.*, immune fitness model) [145,146]. Some expression validation is represented by transcript per million (TPM) in transcriptome if the former prediction of neoantigen candidates is performed in genome data. Validation can also be represented by peptide search in proteome database or cohort-specific proteome datasets provided by users, using basic local alignment search tool-protein (BLASTP) or Peptide Match [147,148]. Tools like pVAC-Seq, ProGeo-neo, TruNeo, and TSNAD v2.0, could quantify and assign values to some aforementioned criteria, integrating HLA-binding affinity and stability with assigned weights to provide comprehensive scoring systems [52,61,67,142]. These algorithms, together with peptide performance predictors, are classified in Table 1 and featured in constructed data portals (Figure 3).

## Data resources of tumor neoantigens

To date, plenty of neoantigens from different origins and across cancer types have been discovered and collected in databases based on the development of both computational and experimental strategies. Herein, we summarize these databases, including NeoTCR, IEDB, TSNAdb v2.0, dbPepNeo2.0, NEPdb, Neodb, TSNAdb, dbPepNeo, GNIFdb, cancer antigens database (CAD) v1.0, small peptides encoded by ncRNA from cancer patients (SPENCER), and TransLnc to provide references and resources for neoantigen search [53,95,96,142,149–155] (**Table 2**). These databases share some similar methods and provide general function modules, such as searching for HLAs and genes, browsing neoantigens, and downloading databases. Some databases also developed specific methods or modules. For example, dbPepNeo2.0 incorporates DeepCNN-Ineo — a deep learning model for predicting the immunogenicity of neoantigens — to aid users in identifying their own candidate neoantigens [151]. GNIFdb and CAD provide the visualization of the genome across cancer subtypes and MHC–peptide structures, respectively, to help users get a better understanding of sequences and products of different neoantigens [155,156]. GNIFdb also provides a list of non-antigens, including 482,109 peptides derived from 100 non-antigens by 9-mer sliding window, which would save users’ time by excluding these non-antigen peptides [155].

**Table 2:** Resources of tumor neoantigens

Database	Algorithm and tool	Neoantigen origin	Link	Ref.
IEDB	NN-align [188], SMM-align [186], TepiTool [204], NetCTL [205], NetCTLpan [189], MHC-NP [206], MHCII-NP [207], DockTope [208], PepX [209]	Integrating multiple resources and origins	https://www.immuneepitope.org/	[144]
TSNAdb v2.0	NetMHCpan-4.0 [116], DeepHLApan [210], MHCflurry [140]	SNVs/InDels, gene fusions	https://pgx.zju.edu.cn/tsnadb/	[142]
dbPepNeo2.0	NetMHCpan-4.1 [94], NetMHCIIpan-4.0 [94], ProGeo-neo [61], DeepCNN-Ineo, BLAST [147], INeo-Epp [211]	SNVs/InDels, gene fusions	http://118.31.70.55/dbPepNeo2.0/	[151]
NeoTCR	UMI-tools [212], Trimmomatic [213], MiXCR [145], Echarts [214], VDJdb [215], McPAS-TCR [216]	SNVs/InDels, gene fusions, AS	ttp://neotcrdb.bioxai.cn/home	[149]
NEPdb	NetMHCpan-4.0 [116], HLAthena [217]	SNVs/InDels	http://nep.whu.edu.cn/	[152]
Neodb	IEDB [144], NetMHCpan-4.0 [116], NetMHCstabpan [218], MHCflurry [140], MHCnuggets [203], NetChop [219], NetCTLpan [180], Seq2Neo-CNN [69], DeepImmuno [195], PRIME2.0 [220]	SNVs/InDels	https://github.com/XSLiuLab/Neodb	[153]
TSNAdb	NetMHCpan-4.0 [116], NetMHCpan-2.8 [177]	SNVs/InDels	http://biopharm.zju.edu.cn/tsnadb/	[142]
dbPepNeo	NetMHCpan-4.0 [116], ProGeo-neo [61], INeo-Epp [211]	SNVs/InDels	http://www.biostatistics.online/dbPepNeo/index.php	[154]
NeoPeptide	NA	SNVs/InDels	https://github.com/lyotvincent/NeoPeptide	[221]
GNIFdb	protFP [157], BLOSUM indices [158], Cruciani Properties [159], FASGAI [222], MS-WHIM [157], Kidera Factor [223], stScales [224], T-scale [222], zScales [225], VHSE-scales [226], AA distribution [157], phisical and chemical Properties [160], neoDL [227], NES [228]	SNVs/InDels	http://www.oncoimmunobank.cn/index.php	[155]
CAD v1.0	NetMHC [138], NetMHCpan-4.1 [94]	SNVs/InDels	http://cad.bio-it.cn/	[156]
SPENCER	CPAT [92], ViennaRNA [229], NetMHCpan-4.0 [116], NetMHCstabpan [143]	lncRNAs	http://spencer.renlab.org	[96]
TransLnc	NetMHCpan-4.1 [94], NetMHCIIpan-4.0 [94], OrthoMCL [93]	lncRNAs	http://bio-bigdata.hrbmu.edu.cn/TransLnc/	[95]

*Note*: lncRNA, long non-coding RNA.

Some database websites provide tools predicting affinities between neoantigens and HLAs (Figure 2; Table 2), such as NetMHCpan-4.0 in NEPdb, TSNAdb, Neodb, dbPepNeo, and CAD [53,116,142,152–154,156]. Additionally, some databases incorporate aforementioned algorithms and/or tools capable of predicting neoantigens, such as Neodb incorporating Seq2Neo and dbPepNeo incorporating ProGeo-neo [61,69,153,154]. Crossing and merging of algorithms, tools, and databases provide abundant resources for neoantigen discovery and exploration.

Among the databases, IEDB gathered antigen data from multiple resources and has been widely used in neoantigen predicting tools, such as IRIS, which identifies AS-derived neoantigens [104,150]. Multiple analysis tools targeting different aspects of neoantigen immunogenicity are presented by IEDB, in which users could choose the preferred tools for analyses. Another pan-cancer database, TSNAdb, incorporated predicated neoantigens derived from somatic mutations across 16 tumor types with 7748 tumor samples and provided shared neoantigens based on recurrent mutations among cancer types and prevalent HLA alleles [53]. The updated version, TSNAdb v2.0, also provided information on mutation types and experimental validations [142]. Unlike the broad information incorporation, GNIFdb specifically gathered neoantigens in glioma while providing a more detailed analysis for neoantigen selection, such as protFP descriptor, blocks substitution matrix (BLOSUM) indices, Cruciani properties, and physical and chemical property predictions [155,157–160]. SPENCER and TransLnc provide predicted neoantigen sequences derived from translative long non-coding regions [95,96]. Incorporating predicted and/or validated neoantigens into these data portals can aid researchers in discerning cancer-specific antigens. The comparative analysis between newly identified peptides and existing epitopes within these resources may also provide a more elucidating approach for exploring novel treatment.

## Conclusion and perspectives

In conclusion, researchers have made remarkable advancements in neoantigen discovery and prediction in recent years, propelled by techniques like high-throughput sequencing, multi-omics profiling, and innovative computational tools and algorithms leveraging machine learning and deep learning [161]. The expansion of these tools and algorithms enriches our understanding of the immunogenic landscape associated with diverse origins of neoantigens, including somatic mutations (SNVs/InDels and gene fusions), circRNAs, AS, RNA editing, and polymorphic microbiomes. Most current tools focus on peptides derived from one or two origins, such as TIminer (SNVs/InDels) and NeoFuse (gene fusions) [60,77]. However, only a few tools have integrated functions to predict peptides from multiple origins, such as LENS (SNVs/InDels, gene fusions, AS, and viruses) and ScanNeo2 (SNVs/InDels, gene fusions, and AS) [63,162]. Additionally, there is a lack of objective benchmarking in this field, which may make the selection of tools, algorithms, and data resources difficult for users. In this review, we conclude some of the characteristics, while a more detailed benchmark work is needed.

As for more specific predictions, existing tools have showcased adaptability in predicting neoantigens derived from RNA editing and polymorphic microbiomes. However, intact pipelines or novel algorithms predicting such neoantigens require further development. With regard to exogenous microbiome, the integration of these neoantigens with host immune profiling remains unexplored. Additionally, considering peptide homology with human proteins, it remains unclear whether such homology will affect later immune responses [163]. Advanced algorithms like deep learning have not yet been widely integrated into neoantigen prediction despite pioneering tools like TruNeo [67]. Perhaps integrating such algorithms could help improve the predictive accuracy and sensitivity of current algorithms. In summary, optimizing tools and algorithms for predicting these specific tumor neoantigens contributes to expanding the application of this field. In addition, the translation potentials of some regions formerly known as non-coding regions have recently been explored. Databases and resources are gathering predicted neoantigens from such regions [95,96], while the predicting processes were not integrated into user-friendly tools to make neoantigen discovery in other cancer datasets more efficient.

The consideration of incorporating different sequencing data is usually based on the origins of neoantigens. For example, the detection of somatic mutations requires genome data like whole-genome sequencing (WGS) or whole-exome sequencing (WES), while the detection of AS requires RNA-seq data. Expression validation for neoantigen prioritization is a great example of combining multi-omics data. Furthermore, proteome data would be the major contributor to improving reliability and decreasing false discovery rates for confirming the existence of translated peptides. Integrative multi-omics data, including genome, transcriptome, and proteome, could improve the accuracy and reliability of neoantigen predictions. For example, nextNEOpi utilizes WES/WGS data to predict mutations and HLA types. It also utilizes RNA-seq data to predict gene fusions and to reconfirm the HLA types predicted by WES/WGS, which further improves the accuracy of later affinity prediction [66]. The combination of multi-omics data not only serves data-specific prediction needs but also refines the accuracy of predictions that could obtain different sources of data. Additionally, the enhancement of resolution based on single-cell RNA sequencing (scRNA-seq) may provide a better understanding of neoantigens. Recent studies have shown the utility of scRNA-seq, *i.e.*, Smart-seq2, in neoantigen prediction [107,164]. For example, researchers tried to use SNAF to identify neo-junctions that could become neoantigens in terms of tumor cells in multiple cell types from both bulk RNA-seq and scRNA-seq data [107]. Specific tools or algorithms in mining neoantigens from scRNA-seq data still need further exploration.

Furthermore, higher-throughput exploration based on experiments is required to fully identify the functions of neoantigens and provide reliable background on *in silico* predictions afterward. Database resources incorporating experimental details like TSNAdb v2.0 also call for more combinations of computational predictions and experimental validations [142]. With multi-omics data support, integrated predictors, improved algorithms, and experimental validations, more tumor neoantigens with potential clinical functions will be identified to advance drug discovery and develop novel therapeutical strategies.

## CRediT author statement

**Yunzhe Wang:** Writing – original draft, Writing – review & editing, Visualization, Data curation. **James Wengler:** Writing – original draft. **Yuzhu Fang:** Visualization, Data curation. **Joseph Zhou:** Writing – review & editing. **Hang Ruan:** Writing – original draft. **Zhao Zhang:** Funding acquisition, Supervision, Writing – review & editing. **Leng Han:** Conceptualization, Supervision, Project administration, Writing – review & editing. All authors have read and approved the final manuscript.

## Competing interests

The authors have declared no competing interests.
